# Vascular Physics: Explaining the Nature of Escape Veins and When to Use Endovascular Ligation

**DOI:** 10.7759/cureus.3354

**Published:** 2018-09-24

**Authors:** Taylor S Harmon, James Cunningham, Abdur R Khan, Erik Soule, Jerry Matteo

**Affiliations:** 1 Interventional Radiology, The University of Texas Medical Branch, Galveston, USA; 2 Interventional Radiology, University of Florida Health, Jacksonville, USA; 3 Interventional Radiology, Memorial Medical Center, Modesto, USA; 4 Interventional Radiology, University of Florida College of Medicine, Jacksonville, USA

**Keywords:** electrical circuit, exclusion, venous blood flow, av fistula, endovascular, ligation, pressure gradient, hemodialysis, interventional radiology, escape veins

## Abstract

Small branching veins that arise from the venous outflow of surgical arterial-venous fistulas (AVFs) are frequently seen during fistulograms performed to evaluate for poorly functioning AVFs. It is hypothesized that the presence of escape veins can decrease the performance of native AVFs during hemodialysis by diverting flow. Though interventional methods for exclusion of escape veins are effective, the mechanism of disruption these small branching vessels cause on flow through AVFs is unknown. Furthermore, an objective method for identifying escape veins that cause significantly diminished venous flow has not been defined. The following describes the detrimental nature of escape veins using tenants of physics and electrical circuitry. Subsequently, the proceeding study shows the identification of small branching escape veins in patients during fistulography. Intravascular pressure measurements were obtained proximal and distal to the ostium of the offending collaterals in these patients. Escape veins causing a pressure gradient of at least 5 mmHg were treated, and pressure measurements were repeated following intervention. The patients were entered into a database and hemodialysis blood flow rates were monitored to determine if escape vein intervention increased AVF performance.

## Introduction

Escape vein collaterals are quite common after the creation of native arterial-venous fistulas (AVFs) and many times unavoidable, as the surgical exposure performed to create the arterial-venous (AV) anastomosis does not always allow the surgeon to identify these venous side branches [1]. The consequences of escape veins are thought to be the diversion of flow from the main channel of the fistula, as well as disruption of laminar flow, leading to decreased performance during hemodialysis (HD) [2]. In newly created native fistulas, escape veins can prevent equal pressurization across the anastomosis and venous outflow, leading to immature arterialization of the venous tract [3]. Furthermore, escape veins are often inadvertently punctured during hemodialysis access, which can cause poor flow through the dialysis circuit, leading to prolonged or incomplete treatment times [4]. It is not uncommon to accidentally cannulate these small branching veins, which can also cause post-hemodialysis hemorrhage and infiltration into the extremity [5].

Small branch veins arising from a native AVF can redirect laminar venous flow, and is analogous to the diversion of current or resistance in an electrical circuit. Likewise, properties of electrical circuitry including voltage and current can explain the effects that escape veins have on laminar venous flow in a native AV fistula. For hypothetical purposes, if we consider blood flow analogous to current, escape veins analogous to resistance, and AV pressure gradient analogous to voltage, siphoned blood flow from escape veins can be observed and analyzed using tenants of physics and electrical circuitry. In order to fully comprehend when escape veins require interventional management, it is important to first understand how escape veins can deter venous flow. Only then, a standardized criterion for excluding these small branching veins can be established to improve the efficiency of hemodialysis AVFs.

## Materials and methods

**Figure 1:**
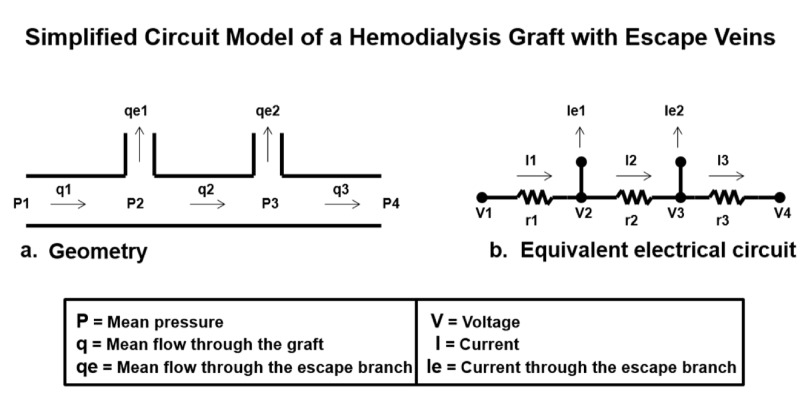
Simplified circuit model of a hemodialysis graft with escape veins. A simplified circuit model of a hemodialysis venous outflow is shown on the left labeled geometry. (a) An equivalent electrical circuit on the right side is labeled. (b) The schematic illustration compares pressure and flow (for the venous outflow) to voltage and current (for the electrical circuit).

By applying Kirchhoff’s current law, Ohm’s law, and Kirchhoff’s voltage law, the resistance to total venous flow caused by escape veins can be calculated. Furthermore, the improvement in total venous flow by the exclusion of escape veins can be determined. The following will explain the clinical manifestation of escape veins, by first describing the analogous nature of blood flow and current. Comparing schematics describing the physics of electrical circuity and venous blood flow reveals many similarities (Figure 1).

Small branching veins can function as “escape” currents in a circuit, where they divert physiologic venous flow from a native AVF. Total venous blood flow can be calculated in Kirchhoff’s current law, where any escape currents that divert blood flow from a native AVF can be subtracted from the total venous flow. As in a closed system electrical circuit, current entering and leaving a junction is conserved:

$$\large I_{Total} = I_{1} + I_{2}...I_{n}$$

**Figure 2:**
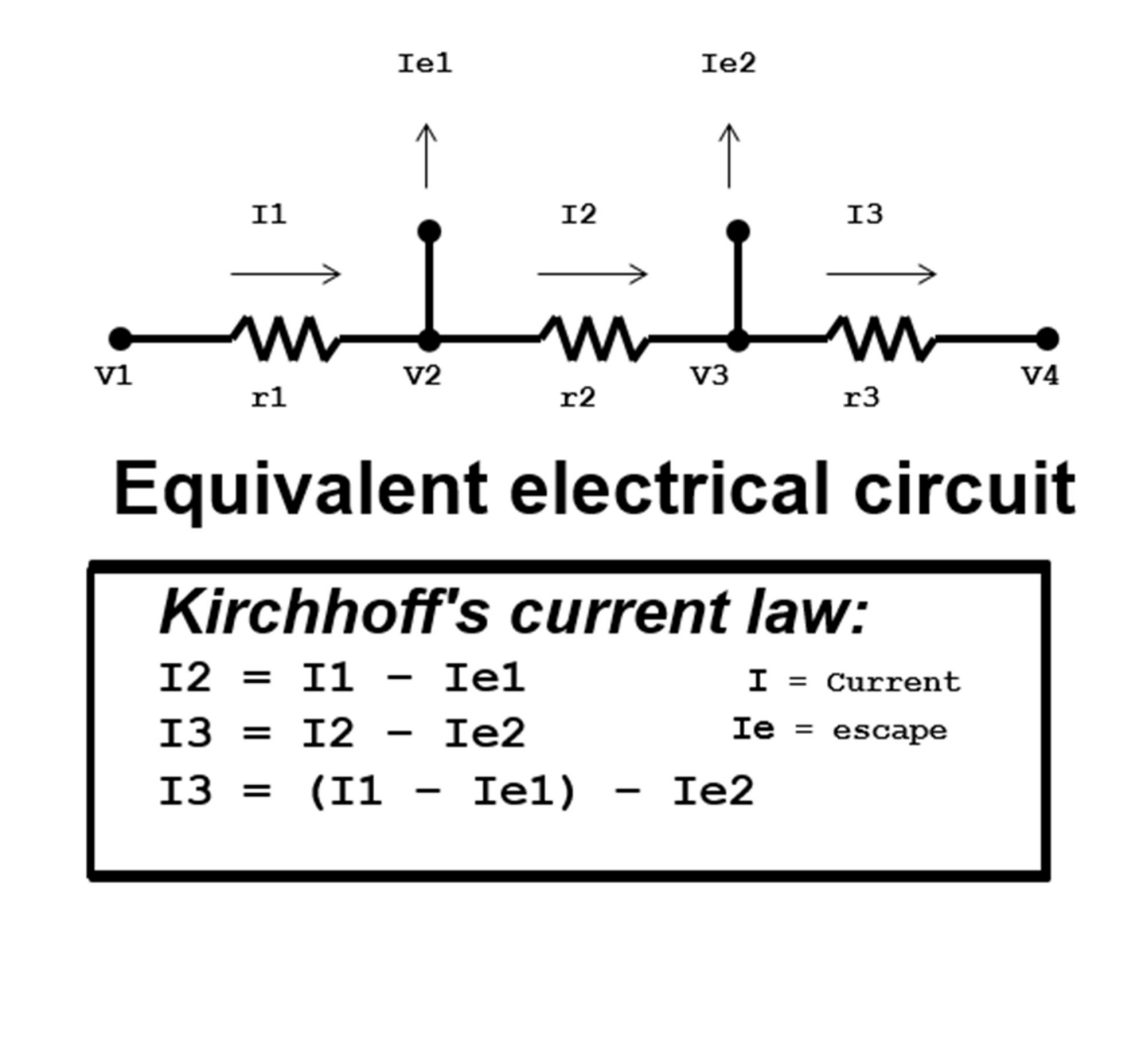
Kirchhoff's current law. A schematic illustration of the electrical circuit is shown, and applies Kirchhoff's current law in mathematical terms to what happens during escape currents labeled Ie1 and Ie2. “I” represents the current of the electrical circuit, “V” represents the voltage at particular points in the circuit, and “r” represents resistors of the circuit.

Kirchhoff’s current law demonstrates that at a separating junction of two or more circuits, current will be evenly distributed amongst them, but subtracted from the total current. If the branched circuits are rejoined at a subsequent junction, as long as there is no resistance, the sum of the currents from each branched circuit should be equal to the total circuit current [6]. At a separating junction where a circuit parts to form two or more circuits, the current of the circuit is distributed evenly between the circuit paths. Though Kirchhoff’s current law does not account for resistance in a circuit, it demonstrates that in a closed system, energy is conserved and the total current in a system can be distributed evenly, whether resistance is present or absent [7]. Additionally, if the branched circuits are never to join again, the current will remain evenly distributed amongst the branched circuits, lessening the branched circuit currents distal to the separating junction. In the case of small branching escape veins, equivalent to branched circuits, the measured laminar venous flow (or total venous current) will decrease distal to the escape vein separating junction (Figure 2).

The decrease in venous flow (or current) is proportional to the flow potential difference, synonymous with voltage, and can be further calculated using Ohm’s law. Ohm’s law states that current is directly proportional to the distance between two points proximal and distal in a circuit, otherwise known as electrical potential difference or voltage [8]. This introduces an indirectly proportional constant, the resistance, which draws current from the circuit:

$$\large Voltage=(Current)(Resistance)$$

$$\large V=IR$$

**Figure 3:**
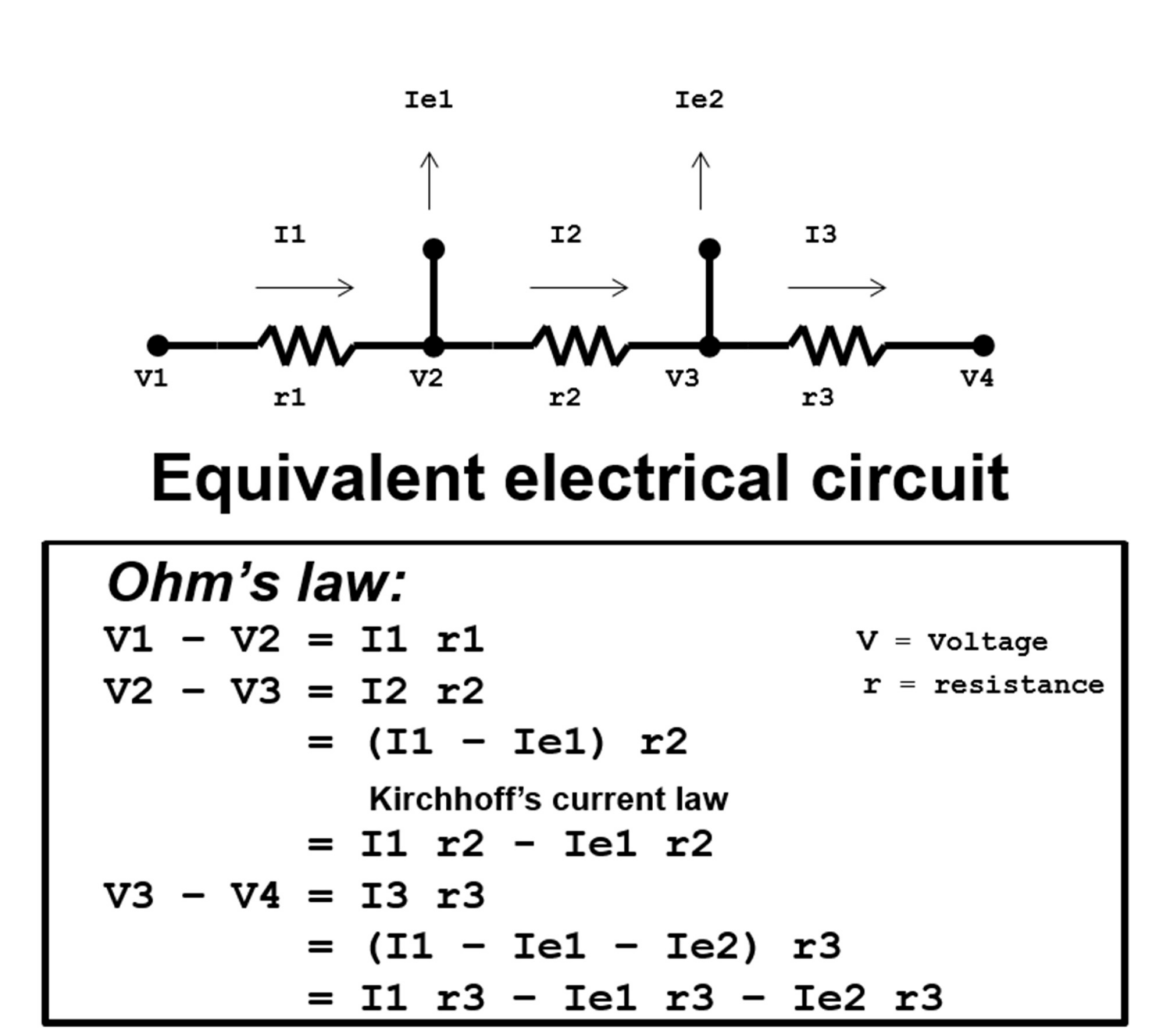
Ohm's law. A schematic illustration of the electrical circuit is shown, and applies Ohm's law in mathematical terms to what happens during escape currents labeled Ie1 and Ie2. “I” represents the current of the electrical circuit, “V” represents the voltage at particular points in the circuit, and “r” represents resistors of the circuit.

The voltage, or flow potential difference, can be calculated across escape veins as would be the case in an electrical circuit with resistors [9]. As current moves across resistors in a circuit, the current and electrical potential decrease [10]. In parallel, the decrease of total venous flow across escape veins is equivocal to the decreased flow potential difference. Examples of Ohm’s law as applied to laminar venous flow are as follows (Figure 3):

Notice that Ohm’s law accounts for the decrease in voltage where electrical potential difference is decreased either across a resistor, or when the total circuit current is decreased by a branching circuit (Ie1, Ie2). The resistance of a circuit decreases the electrical potential difference across it, resulting in decreased total circuit current. Similarly, escape veins decrease the total venous flow (analogous to total circuit current) across native AVFs, resulting in AVF malfunction.

**Figure 4:**
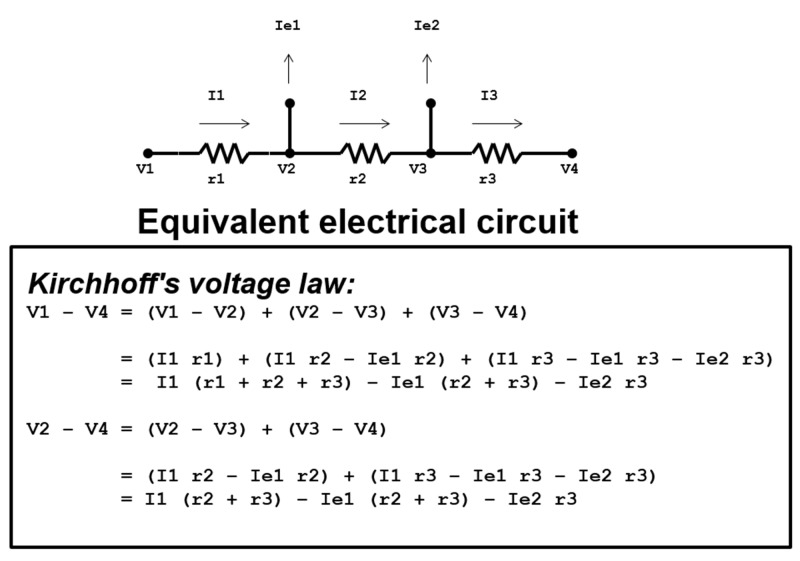
Kirchhoff's voltage law. A schematic illustration of the electrical circuit is shown, and applies Kirchhoff's voltage law in mathematical terms of how voltage from V1 to V4 diminishes secondary to relinquished energy that has escaped via Ie1 and Ie2. “I” represents the current of the electrical circuit, “V” represents the voltage at particular points in the circuit, and “r” represents resistors of the circuit.

As in Kirchhoff’s current law, Kirchhoff’s voltage law demonstrates conservation of electrical potential difference in a closed circuit. In correspondence with Kirchhoff’s current law, Kirchhoff’s voltage law further demonstrates that two or more joining circuits at a junction point will have additive currents and voltages [11]. Since current is directly proportional to voltage and inversely proportional to resistance as demonstrated by Ohm’s law, both the current and voltage will decrease through a circuit resistor. With this understanding, the decreased electrical potential difference across a circuit resistor can be calculated and synonymously applied to the decreased flow potential difference across escape veins (Figure 4).

In each subsequent resistor in a circuit, the total voltage and current decreases, which reflects the same changes in venous blood flow; after each subsequent escape vein, the total venous flow and flow potential difference are decreased [12]. In the event escape current (Ie1 or Ie2) equals zero, meaning either the escape vein does not exist, has been treated with intervention, or does not siphon enough flow to be clinically significant, the voltage and current will remain unaffected. In an electrical circuit, if there are not any separating junctions, and assuming the resistance in the total circuit is zero, then the most proximal voltage (V1) will be equivalent to the most distal voltage (V4) [13]. In a venous outflow system across a native AVF, if we assume V4 (corresponding to mean systemic venous pressure) is constant, it can be concluded that V2, V3, and V4 (corresponding to post-interventional pressures) will increase and equilibrate with V1 if we render Ie1 and Ie2 (escape veins) to zero. As in the case of total venous flow through a native AVF with developed branching escape veins, if siphoned flow through the escape veins is negligible, the total venous flow and total flow potential difference will not be affected. In practice, the interventional management of escape veins will increase the total venous flow, sustaining the function of the native AVF.

**Figure 5:**
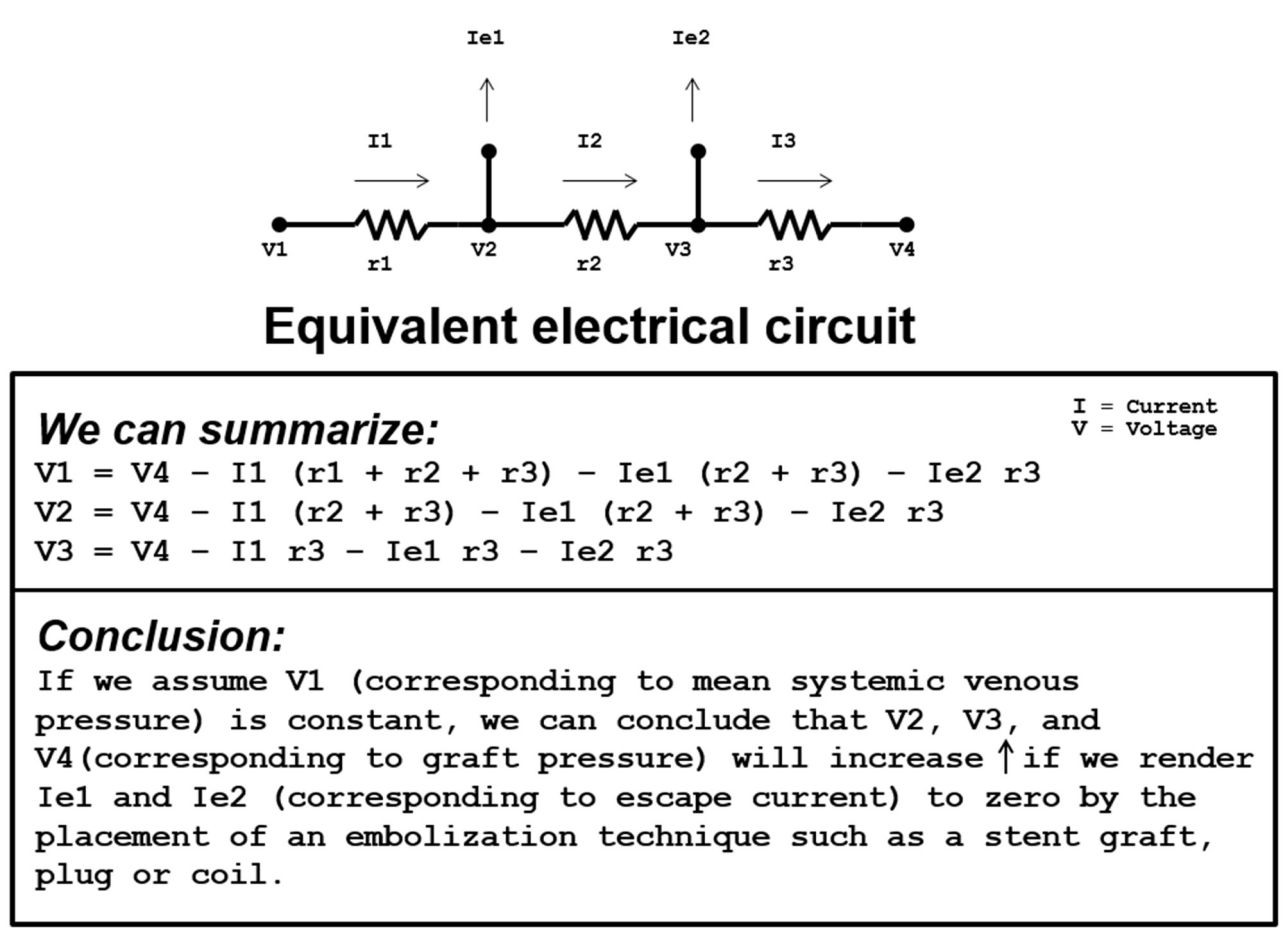
Summation illustration of Kirchhoff's current law, Ohm's law, and Kirchhoff's voltage law. A summation illustration is shown, which demonstrates the electrical circuit as it applies to Kirchhoff's current law, Ohm's law, and Kirchhoff's voltage law. When the escape currents labeled Ie1 and Ie2 (corresponding to "escape veins") are excluded via an embolization technique, V1 = V4. “I” represents the current of the electrical circuit, “V” represents the voltage at particular points in the circuit, and “r” represents resistors of the circuit.

We can summarize the application of Kirchhoff’s current law, Ohm’s law, and Kirchhoff’s voltage law, to elucidate the nature of small branching veins that develop from the placement of native AVFs, sometimes leading to their impairment (Figure 5).

Branching circuits and resistors in a closed electrical circuit model have been shown to be synonymous with escape veins, which divert laminar flow and cause AVF incompetency. Patients with malfunctioning AVFs were observed for changes in total venous blood flow before and after exclusion of escape veins by various interventional methods. These methods were based on operator preference, and the outcomes for each were compared. The operator approaches included embolization with coils, the use of endovascular plugs, or exclusion by covered stent grafts [14]. Intravascular arterial blood pressure gradients across a lesion of greater than 10 mmHg warrant consideration for intervention [15]. This study was designed to identify when small branching veins should be treated. The observation of intravascular pressure differentials proximal and distal to escape veins in patients with native AVF disfunction were recorded and compared in the pre- and post-interventional periods. The threshold pressure gradient for treatment of escape veins in this study was chosen to be greater than or equal to 5 mmHg.

**Figure 6:**
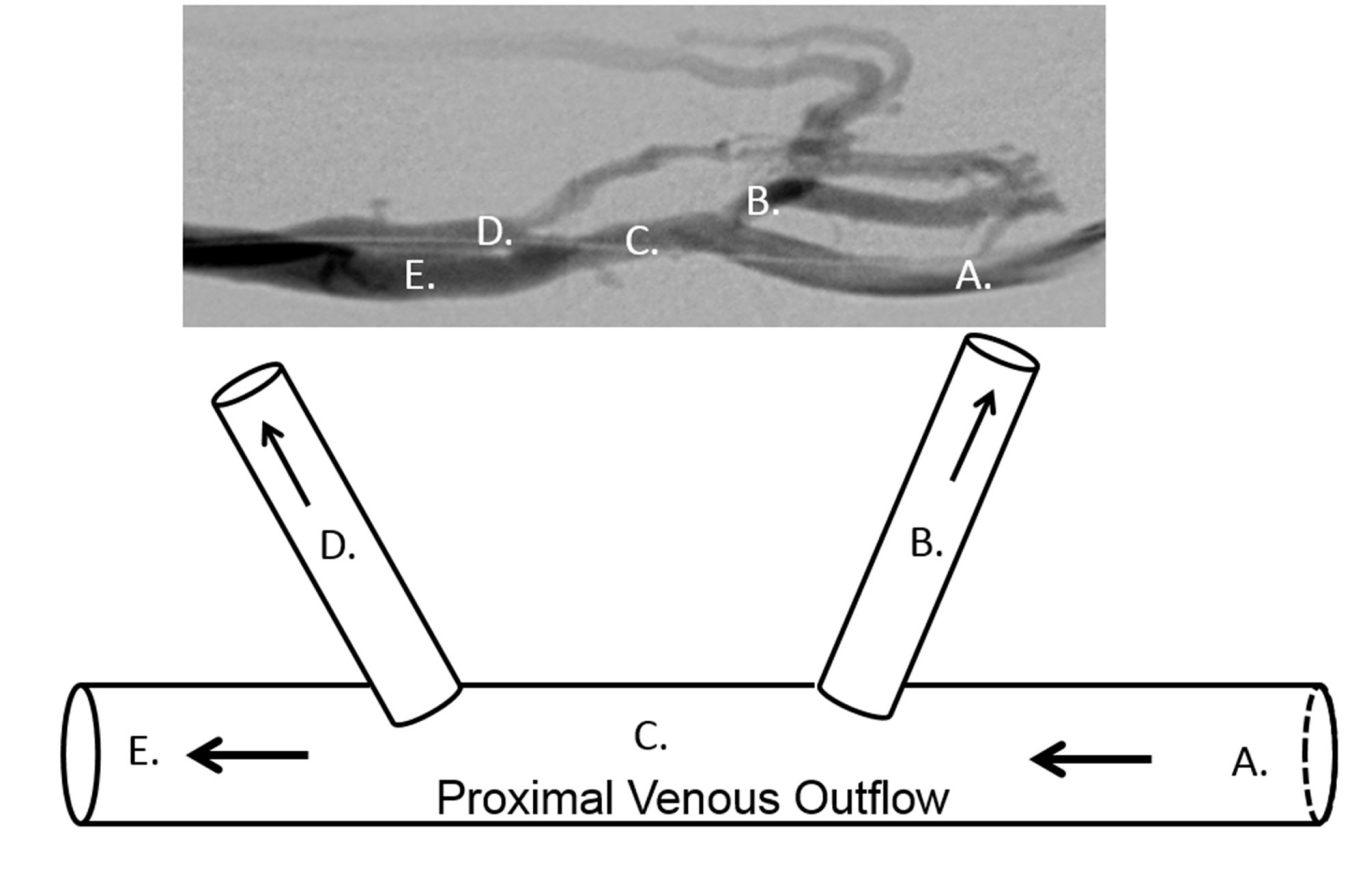
Depictions of illustrated escape veins versus fistulography. The top image demonstrates the proximal venous outflow of a fistula with two escape veins labeled (B) and (D). The bottom image is a schematic illustration model drawing of the above fistulogram. (A) is the venous outflow proximal to the escape veins. (C) is the position of the venous outflow between the escape veins and (E) is the venous outflow just distal to the escape veins.

During a fistulogram procedure performed to evaluate for poor venous flow during hemodialysis, escape veins were identified arising from the main venous outflow tract in 10 patients. For the purposes of explanation, the following portrays the clinical and procedural course of a patient from this study who sustained pre- and post-interventional management for escape vein exclusion (Figure 6).

**Figure 7:**
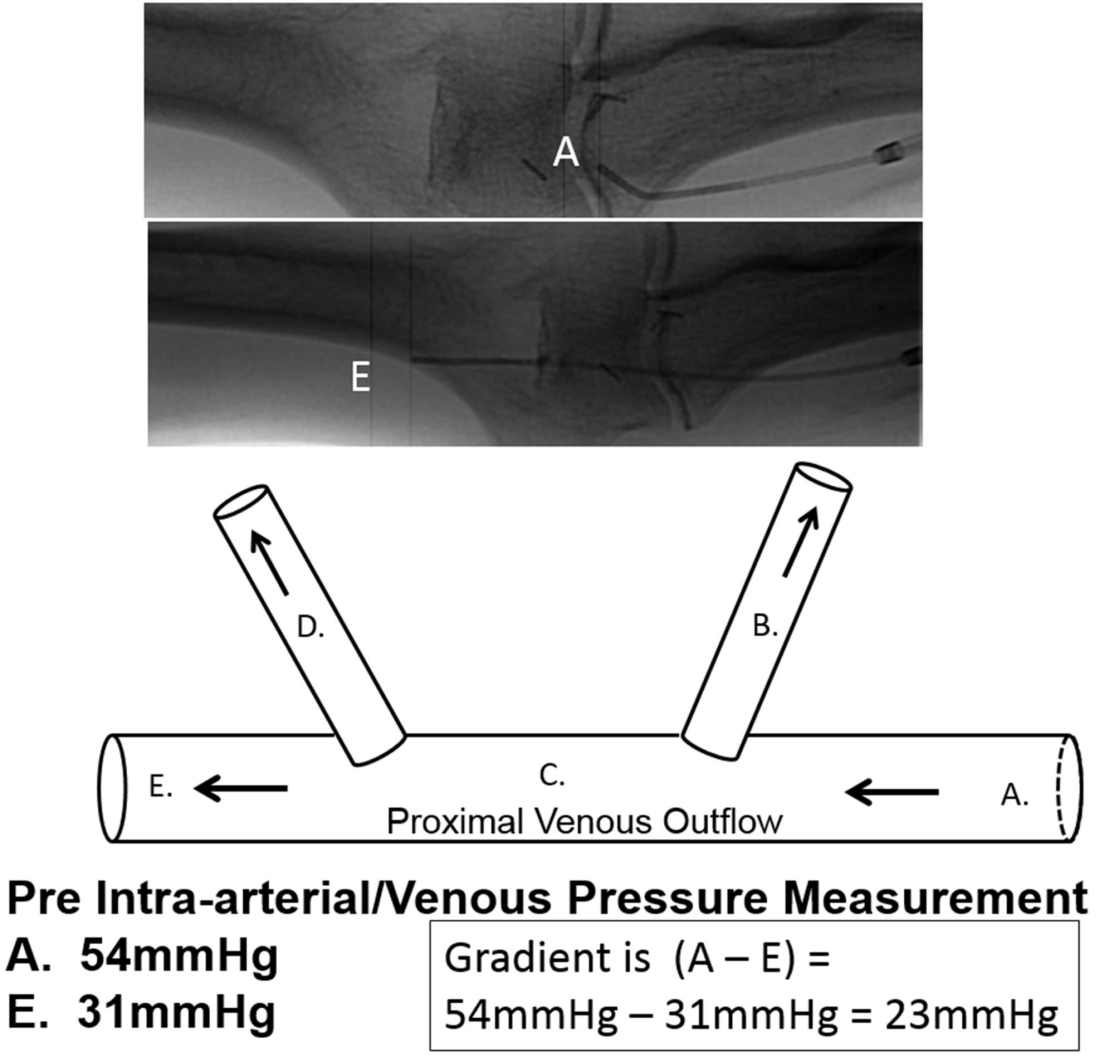
Depiction of illustrated venous pressure gradient across escape veins versus fistulography. The top two images demonstrate the same patient with an end-hole catheter measuring the pressure within the proximal venous outflow proximal to the escape veins (top image labeled A) and the catheter positioned just distal to the escape veins (middle image labeled E). The bottom image is a schematic illustration model drawing of the above fistulogram. The pressure for position (A) is 54 mmHg and the pressure for position (E) is 31 mmHg for a pressure gradient of 23 mmHg.

Pressure measurements were obtained within the main venous outflow at points immediately proximal and distal to each escape vein ostium (Figure 7).

**Figure 8:**
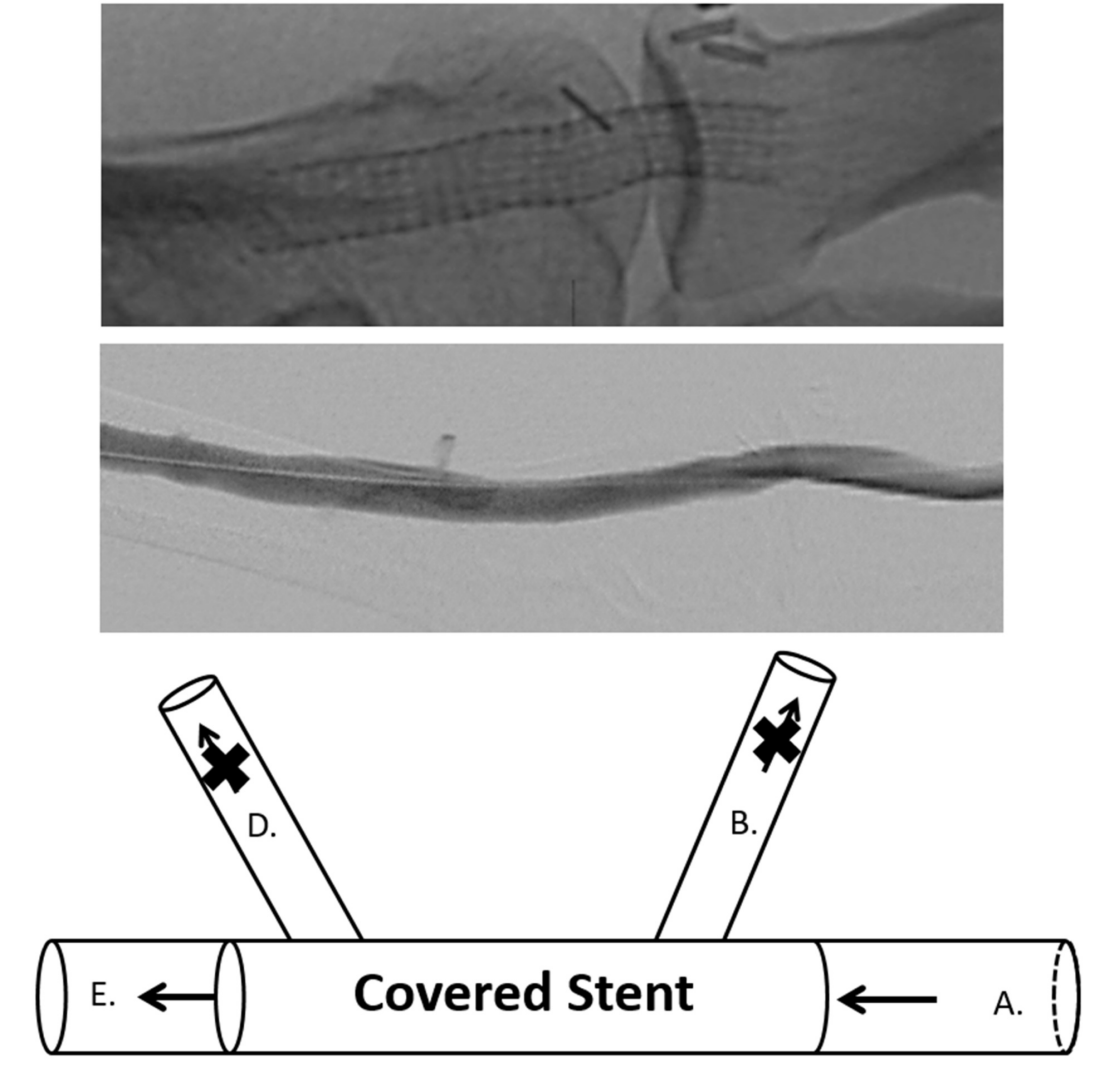
Depiction of illustrated escape veins that have been excluded with a covered stent graft versus fistulography. The top image shows a fistulogram of a covered stent graft across the previously identified escape veins used for exclusion. The middle image shows venous outflow of the fistula through the stent with the previously identified escape veins excluded. The bottom image is a schematic illustration model drawing of the above fistulogram showing the covered stent positioned for exclusion of the escape veins.

The pressures were measured using a 5 French end-hole Berenstein catheter (Boston Scientific, Natick, MA) which was attached to a Namic perceptor manifold device (Navilyst Medical, Inc., Marlborough, MA). Patients with a gradient of 5 mmHg or greater across an escape vein ostium were then treated. The escape veins were treated using a variety of exclusion methods including: Viabahn (W.L. Gore, Flagstaff, Arizona) covered stent grafts, Amplatzer plugs (St. Jude Medical, Plymouth, MN), or Tornado embolization coils (Cook Medical, Bloomington, IN). Though the following schematic depicts a patient who received a covered stent graft for escape venous exclusion, the other aforementioned methods were also used interchangeably in this study, depending on the operator (Figure 8).

**Figure 9:**
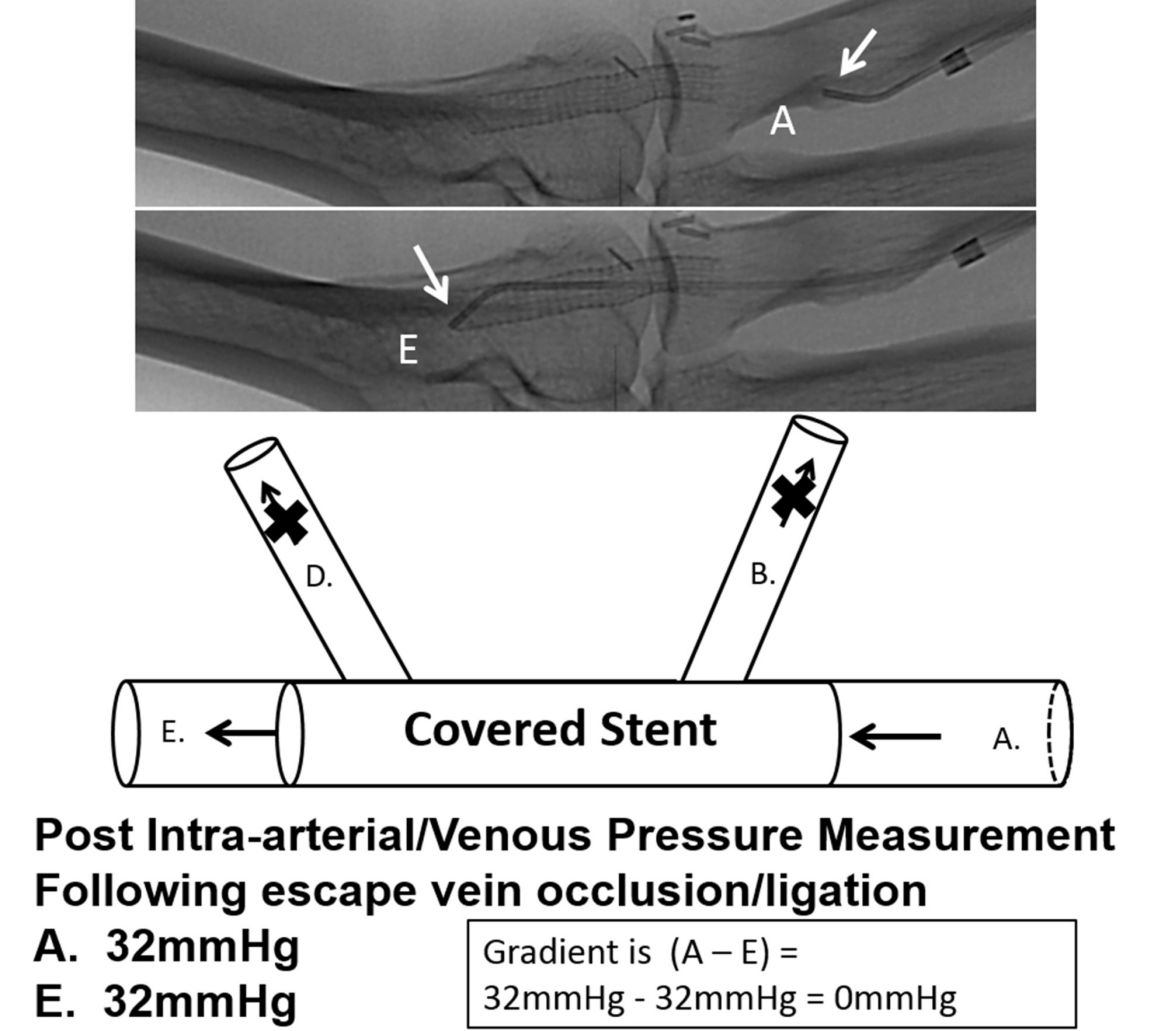
Measured venous pressure gradient of post-interventional excluded escape veins illustrated as a model versus fistulography. The top two images demonstrate the same patient with an end-hole catheter measuring the pressure within the proximal venous outflow proximal to the escape veins (top image labeled A), and the catheter positioned just distal to the escape veins (middle image labeled E) following exclusion with a covered stent graft. The bottom image is a schematic illustration model of the above fistulogram. The pressure for position (A) is 32 mmHg and the pressure for position (E) is 32 mmHg for a pressure gradient of 0 mmHg.

Following exclusion of the escape venous flow by various interventional methods, intravascular pressures were repeated at the same pre-intervention locations (Figure 9).

Ten patients were monitored in pre- and post-interventional periods. These patients were observed for significant changes in total venous blood flow across their native AVFs. The data was successfully compiled and analyzed for the purposes of creating a standardized method for interventional exclusion of clinically significant small branching escape veins.

## Results

The 10 patients presented underwent routine fistulograms to determine the reason for fistula malfunction during hemodialysis. The inclusion criteria for this study consisted of patients that had malfunctioning AVFs or complications of native AVF placement (such as post-interventional bleeding), and/or a venous pressure differential of at least 5 mmHg across any small branching escape vein. These patients’ escape venous tracts were excluded according to operator preference, and their subsequent pressure differentials were measured during a post-interventional period. The patients had prominent escape veins arising from the main venous outflow track of their native AVFs, with pressure differentials that ranged from 5 mmHg to 23 mmHg. The inclusion criteria of the study allowed for two patients (five and nine) to be included in the data set, though they did not have malfunctioning AVFs. Instead, they presented with post-interventional bleeding, qualifying for interventional management (Table 1).

**Table 1:** Escape vein intervention data. The data shows details of the 10 patients involved in the study. Columns two and three show average venous blood flow rates prior to and following intervention, respectively. Column four shows the type of device used for exclusion of the escape veins. Columns five and six show the pressure gradient prior to and following intervention, respectively. Patients five and nine were included in the study because of post-hemodialysis hemorrhage.

Patient Encounter	Average Hemodialysis Flow Rate Prior to Intervention (ml/min)	Average Hemodialysis Flow Rate Following Intervention (ml/min)	Type of Exclusion Intervention	Pressure Gradient Prior to Intervention (mmHg)	Pressure Gradient Following Intervention (mmHg)
1	363	374	Stent	23	0
2	363	374	Coil	7	0
3	316	450	Plug	8	0
4	345	348	Coil	5	0
5	500	500* Post-hemodialysis hemorrhage indication for procedure	Stent	10	0
6	370	400	Stent	23	0
7	400	456	Stent	9	0
8	406	450	Stent	7	0
9	400	400* Post-hemodialysis hemorrhage indication for procedure	Plug	6	0
10	430	500	Coil	6	0

**Figure 10:**
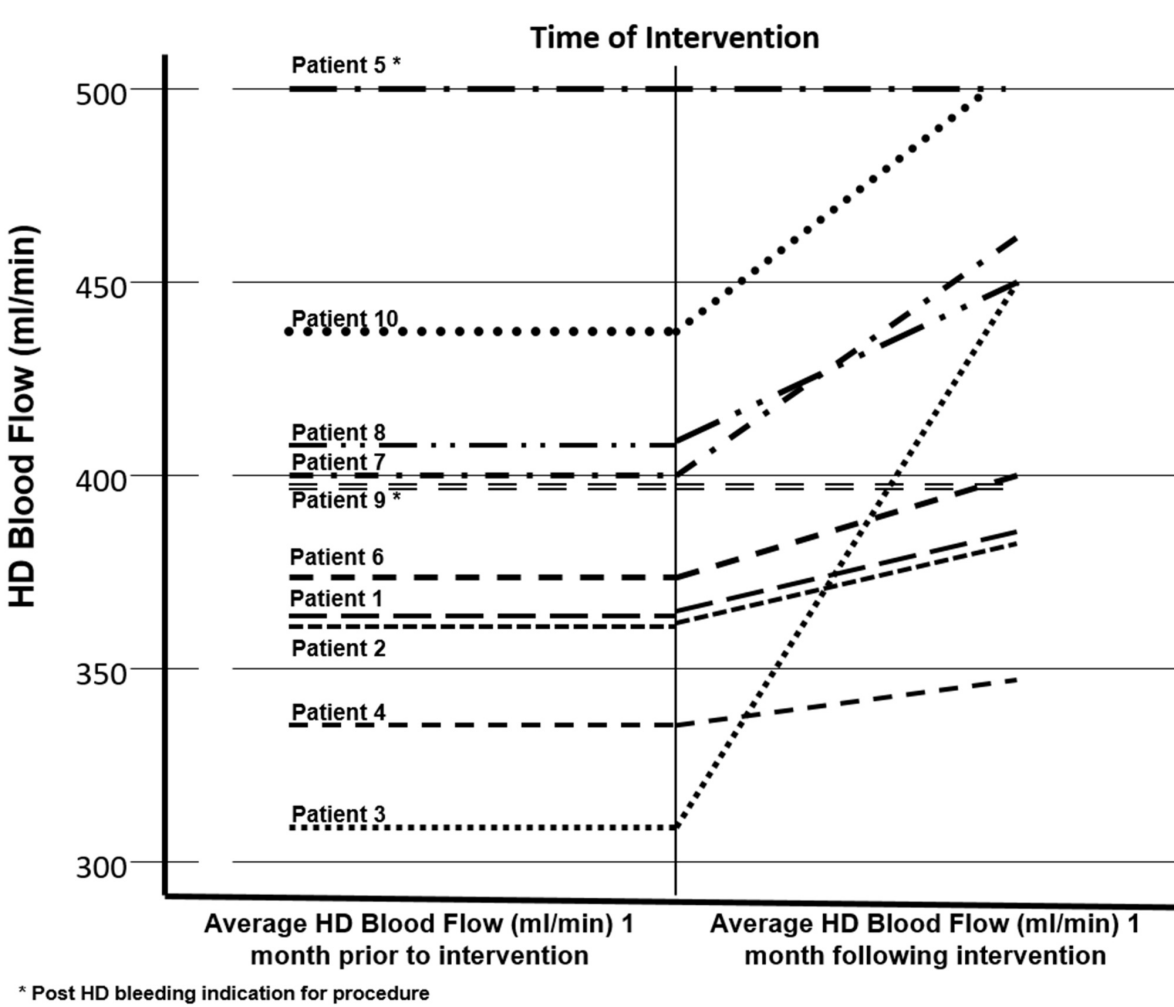
Graphed escape vein intervention data. A graph of the 10 patients plotted showing the average hemodialysis (HD) blood flow rates prior to and following intervention.

Though the option to use a covered stent graft, endovascular plugs, or embolization coils was at the discretion of the operator and dependent on patient vascular anatomy, the outcomes of all interventional methods were equally successful. The flow potential differences in 100% of the patients’ small branching escape veins were reduced to zero, as measured in the post-interventional period. The average hemodialysis blood flow rates were documented up to one month prior to and after intervention. Hemodialysis blood flow rates increased in 100% of the patients treated with malfunctioning native AVFs. All of the patients in the study continued at optimal blood flow rates (Figure 10).

## Discussion

A standardized method for the treatment of escape veins has not been formally described in the literature. The purpose of this pilot study was to determine if escape veins cause a pressure gradient clinically significant enough to divert venous flow from natively placed AVFs, ultimately leading to AVF malfunction. It has now been shown with the results presented in this study that not only do escape vein cause impairment of native AVFs, but interventional methods for salvaging AVFs can be conducted. Furthermore, the mechanism for which escape veins cause inefficient total venous flow through native AVFs has been described for the first time here.

Physics and electrical circuity applied in Kirchhoff’s current law, Ohm’s law, and Kirchhoff’s law describe the analogous relationship between resistors in an electrical circuit, and the small branching veins that siphon blood from the total venous flow in a native AVF. The quantitative and qualitative tenants of escape veins described here, lead to the establishment of an inclusion criteria suitable for the successfully identification and procedural intervention of 10 patients with malfunctioning native AVFs. To our knowledge, the implementation of these guidelines for the interventional exclusion of escape veins with at least a 5-mmHg venous pressure gradient is the first objective criteria that has been characterized.

Moreover, it was proven that any of the various methods described for escape vein exclusion was independent in the success of intervention and substantial increase in total venous flow. Native AVFs in 100% of the patients with malfunctioning AVFs who qualified for interventional management had increases in total blood flow as measured in the post-interventional period.

## Conclusions

Small branching escape veins have been shown to cause inadequate venous flow in patients who rely on life-sustaining hemodialysis. Early intervention in impaired native AVFs as a result of escape venous collateral flow can salvage the fistula and prevent additional complications. This study demonstrates a 100% post-interventional increase in total venous blood flow in patients who met the inclusion criteria for escape venous exclusion. Currently, a standardized protocol for escape venous exclusion does not exist in the literature. However, interventional management as described can now be applied to treatment across any escape vein with a baseline venous pressure gradient of at least 5 mmHg. These findings are attributed to the comprehension of Kirchhoff’s current law, Ohm’s law, Kirchhoff’s voltage law, and the simplified circuit model, where venous blood flow is analogous to current, escape veins are analogous to resistance, and AV pressure gradient is analogous to voltage.
